# Metastatic Prostate Cancer With Reticular Micronodular Opacities of Lung: A Case Report

**DOI:** 10.7759/cureus.42698

**Published:** 2023-07-30

**Authors:** Srikaran Bojja, Nismat Javed, Muhammad Ali Aziz, Ked Fortuzi, Misbahuddin Khaja

**Affiliations:** 1 Internal Medicine, BronxCare Health System, Icahn School of Medicine at Mount Sinai, New York City, USA; 2 Pulmonology, BronxCare Health System, Icahn School of Medicine at Mount Sinai, New York City, USA; 3 Pulmonology and Critical Care, BronxCare Health System, Icahn School of Medicine at Mount Sinai, New York City, USA

**Keywords:** psa level, anti-androgen therapy, micronodular opacities, pulmonary metastases, prostate cancer

## Abstract

Prostate cancer, a common malignancy in males, can metastasize to various sites such as the bone, brain, liver, and less commonly, the lung. Detecting pulmonary metastases presents both diagnostic and therapeutic difficulties. Identifying patients with this condition is crucial for gaining a deeper comprehension of the disease's pathogenesis. In this report, we describe the case of a 64-year-old African American male who exhibited elevated prostate antigen levels and was found to have unique reticular Micronodular opacities in the lungs caused by prostate cancer.

## Introduction

Prostate cancer is one of the diseases with a massive global burden and is primarily prevalent in males [1,2]. The estimated global incidence is about >1,100,000 cases and 300,000 deaths every year [2]. In the US, prostate cancer is the second leading cause of death with about 3.1 million cases diagnosed from 2003 to 2017 [3]. It is more common in older males [2]. Prostate cancer commonly metastasizes to the thorax, kidney, adrenal glands, brain, and retroperitoneum [4,5]. Pulmonary metastases are a relatively uncommon phenomenon with only 4.6% of the cases having such metastases [6]. We present a case report of a patient with metastatic prostate cancer manifesting a unique pattern of metastasis of the lung.

## Case presentation

A 64-year-old African American male with a past medical history of chronic obstructive pulmonary disease (COPD) was following up with his primary care provider for an elevated prostate-specific antigen (PSA) level of 276 ng/mL (Reference value: 0.0-4.0 ng/mL). He was initially referred to urology, however, was lost to follow-up for almost a year. He presented to the Urology clinic complaining of weight loss and worsening back pain. On examination, vitals were within normal limits, and the physical exam revealed an enlarged irregular prostate. Further workup with a needle biopsy of the prostate was discussed, but the patient was undecided about the option.

**Figure 1:**
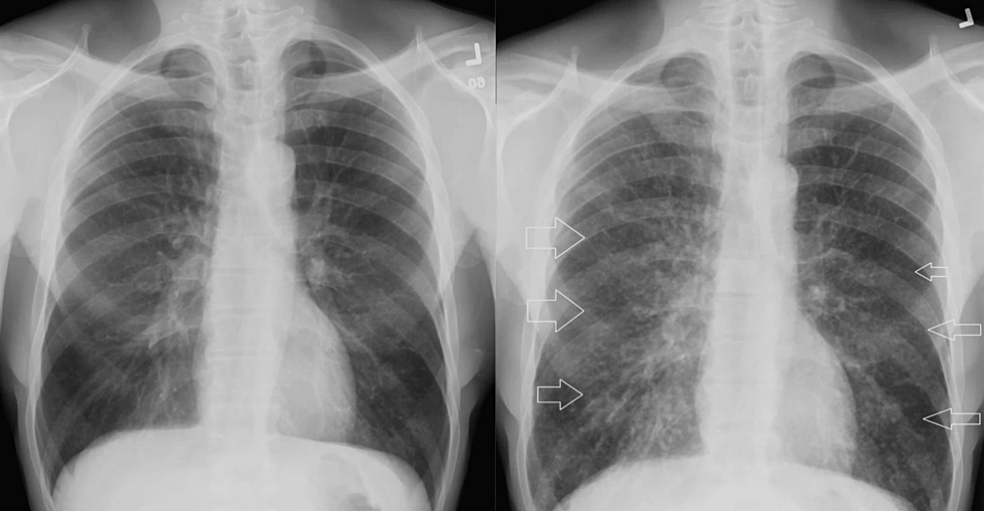
X-ray comparison four years before and at presentation respectively Left-hand side image: Chest X-ray taken four years prior to the presentation was normal. Right-hand side image: Chest X-ray taken at presentation which showed reticulonodular densities (white arrows).

On repeat follow-up, the PSA level had increased significantly to 1116 ng/ml therefore patient was started on Casodex® (bicalutamide). Chest X-ray revealed innumerable reticulonodular densities throughout the lungs which were new compared to previous X-rays (Figure 1).

**Figure 2:**
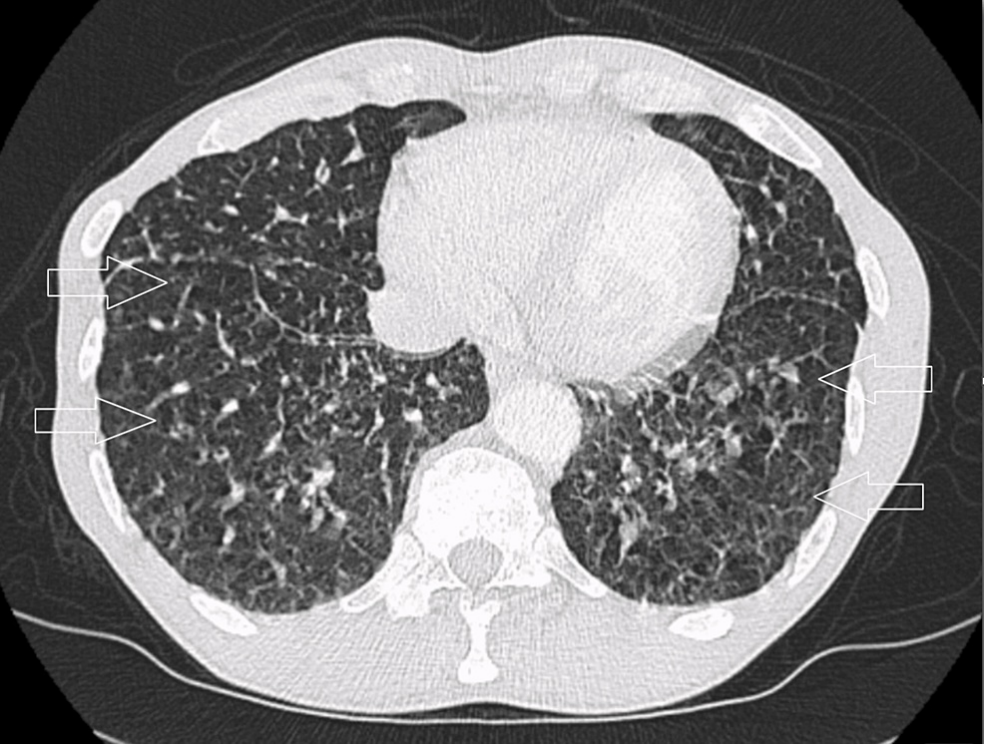
Initial CT chest Initial CT chest revealing diffuse reticular micronodules in both lungs (arrows).

The patient underwent CT of the abdomen and pelvis which revealed prostatomegaly, the mixed lucent and sclerotic appearance of the visualized thoracolumbar spine, iliacs, sacrum, pelvis and femurs, reticulonodular opacities within the right middle and both lower lobes. Technetium 99m-methyl diphosphonate (Tc-99m MDP) whole-body bone scan was done which revealed abnormal distribution of Tc-99m MDP throughout the axial and appendicular skeletal system with multiple foci seen within the pelvis involving the left pubic and ischial bones, right pubic bone and right posterior iliac bone. Additional involvement was seen scattered throughout the thoracic and lumbar spine with additional involvement of the right scapula. He was diagnosed with stage IV prostate cancer and was also started on Eligard®​ (leuprorelin) injections of 22.5mg subcutaneous (SC) every three months. Subsequently, he underwent CT chest for staging which demonstrated bilateral diffuse small reticulonodular opacities in the lungs along with widespread bony metastasis (Figure 2).

**Figure 3:**
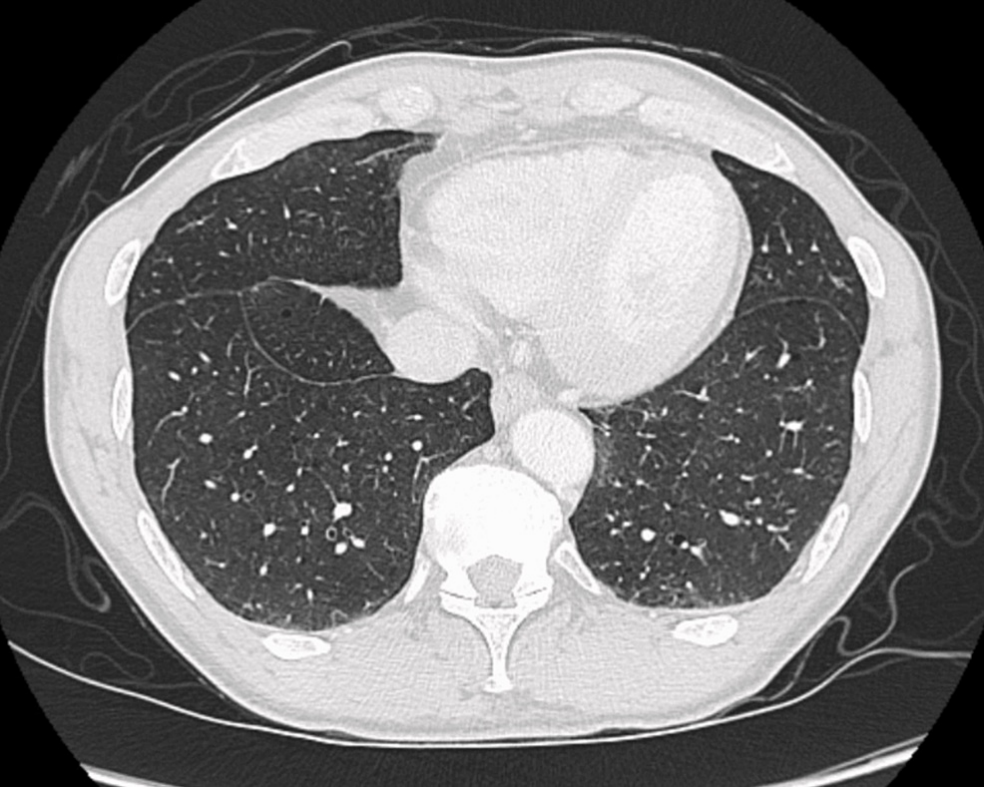
Repeat CT Chest revealing complete resolution of micronodular infiltrates

He followed up with Oncologist and was started on Zytiga® (abiraterone) 1000mg OD (once a day) and prednisone 5mg OD but he refused cytotoxic therapy. He was also planning to get XGEVA® (denosumab) given his bony metastasis. He was also referred to a pulmonology clinic for diffuse bilateral lung micronodules. In addition to pulmonary metastases other differential diagnoses for diffuse micronodular opacities include silicosis, coal workers’ pneumoconiosis, hypersensitivity pneumonitis (cellular HP), sarcoidosis, and miliary infections. They were ruled out as lab investigations were not significant for eosinophilia, sputum for acid-fast bacilli was negative and no prolonged occupational exposure history was noted. After 12 weeks of treatment, repeat imaging was significant for osteoblastic metastases in the thoracic spine and sternum only, but diffuse reticular pulmonary nodules had resolved (Figure 3). There was a significant response to hormonal therapy suggesting the nodular opacities were likely from metastatic prostate cancer. Hence, the patient didn't require any bronchoscopy for biopsy.

On further follow up the patient’s back pain had significantly reduced and PSA had trended down to 4.34 ng/ml in about four months of therapy. He is on hormonal therapy and is following up with oncology. The recent PSA level was <0.01. 

## Discussion

Prostate cancer is commonly seen in older males with median age above 60 years [2]. The underlying pathogenesis involves both hematogenous and lymphatic spread through channels [7]. These metastases are more common in the terminal phases of the disease with concurrent metastases elsewhere including bone and brain [8]. 

There are no specific symptoms suggestive of pulmonary metastasis in prostate cancer. Many patients present with generalized symptoms of weight loss and elevated prostate antigen levels [9]. The presentation of metastases can vary from solitary nodules to widespread involvement [10,11]. The diagnosis depends on appropriate imaging. CT scan might provide additional information [9]. A positron emission tomography-computed tomography (PET-CT) scan can provide visualization of other metastatic lesions [9]. However, in certain cavitary lesions, the yield of these studies is relatively lower [12]. Therefore, decision-making from the overall clinical picture is crucial. 

The management of pulmonary metastases varies depending on the location and type of presentation. Surgical resection is warranted in cases with isolated pulmonary nodules [10,13]. Additionally, lymph node dissection might also be required [10]. Multiple pulmonary nodules require a multidisciplinary approach involving hormonal and radiation therapy. The response of such nodules depends upon patients’ hormone status [14]. In the case of hormone-naïve patients, androgen deprivation offers a promising option with many patients responding favourably [14]. In our case, antiandrogens were utilized owing to bone metastases. Additionally, lung lesions had completely disappeared on repeat scans. 

Pulmonary metastases provide additional information about treatment response to antiandrogens. Patients with pulmonary metastases commonly experience antiandrogen resistance and respond well to androgen escape, that is discontinuing antiandrogen therapy would provide symptomatic improvement [15]. The metastases, however, have not shown a clear role in mortality that is still dependent on the overall stage and complications associated with prostate cancer [11].

## Conclusions

Metastatic prostate cancer uncommonly manifests as diffuse micronodular lung disease, which can be a challenge to identify. Therefore, it is also important for physicians to have a differential diagnosis for reticular micronodular opacities on imaging in mind and rule out any infectious etiologies. Treatment options are limited, and palliative care should be provided to improve the patient's quality of life. Physicians should be aware of this rare manifestation of metastatic prostate cancer in patients with diffuse micronodular lung disease.
